# Enhanced Biosynthesis of Fatty Acids Contributes to Ciprofloxacin Resistance in *Pseudomonas aeruginosa*

**DOI:** 10.3389/fmicb.2022.845173

**Published:** 2022-04-25

**Authors:** Yu-bin Su, Xi-kang Tang, Ling-ping Zhu, Ke-xin Yang, Li Pan, Hui Li, Zhuang-gui Chen

**Affiliations:** ^1^Department of Pediatrics and Department of Allergy, The Third Affiliated Hospital, State Key Laboratory of Bio-Control, Southern Marine Science and Engineering Guangdong Laboratory (Zhuhai), Sun Yat-sen University, Guangzhou, China; ^2^Department of Cell Biology, Ministry of Education Key Laboratory of Tumor Molecular Biology, Guangdong Provincial Key Laboratory of Bioengineering Medicine, Institute of Biomedicine, National Engineering Research Center of Genetic Medicine, College of Life Science and Technology, Jinan University, Guangzhou, China

**Keywords:** *Pseudomonas aeruginosa*, antibiotic resistance, biosynthesis of fatty acids, ciprofloxacin, membrane permeability, metabolomics

## Abstract

Antibiotic-resistant *Pseudomonas aeruginosa* is insensitive to antibiotics and difficult to deal with. An understanding of the resistance mechanisms is required for the control of the pathogen. In this study, gas chromatography–mass spectrometer (GC-MS)-based metabolomics was performed to identify differential metabolomes in ciprofloxacin (CIP)-resistant *P. aeruginosa* strains that originated from *P. aeruginosa* ATCC 27853 and had minimum inhibitory concentrations (MICs) that were 16-, 64-, and 128-fold (PA-R16_CIP_, PA-R64_CIP_, and PA-R128_CIP_, respectively) higher than the original value, compared to CIP-sensitive *P. aeruginosa* (PA-S). Upregulation of fatty acid biosynthesis forms a characteristic feature of the CIP-resistant metabolomes and fatty acid metabolome, which was supported by elevated gene expression and enzymatic activity in the metabolic pathway. The fatty acid synthase inhibitor triclosan potentiates CIP to kill PA-R128_CIP_ and clinically multidrug-resistant *P. aeruginosa* strains. The potentiated killing was companied with reduced gene expression and enzymatic activity and the returned abundance of fatty acids in the metabolic pathway. Consistently, membrane permeability was reduced in the PA-R and clinically multidrug-resistant *P. aeruginosa* strains, which were reverted by triclosan. Triclosan also stimulated the uptake of CIP. These findings highlight the importance of the elevated biosynthesis of fatty acids in the CIP resistance of *P. aeruginosa* and provide a target pathway for combating CIP-resistant *P. aeruginosa*.

## Introduction

*Pseudomonas aeruginosa* is an opportunistic pathogen that causes nosocomial infections, especially in immunosuppressed patients (Pang et al., 2019). The bacterium is characterized by intrinsic and acquired resistance to multiple antibiotics, i.e., bacterium showing resistance to at least three or more classes of antibiotics. Among the classes of antibiotics recommended for testing, ciprofloxacin (CIP) and levofloxacin (LEV) of fluoroquinolones are included (Horcajada et al., 2019). Recently, CIP has been used extensively to treat a wide range of infections caused by *P. aeruginosa*, which is linked to the proportion of CIP-resistant *P. aeruginosa* isolates and is rapidly increasing (Rehman et al., 2019). A strong correlation between increased uses of CIP with increased prevalence of CIP-resistant strains has been determined (Rose et al., 2014). Therefore, understanding the mechanism of CIP resistance is especially important to control CIP-resistant *P. aeruginosa*.

It has been documented that CIP resistance can arise through the acquisition of mutations in genes encoding the target proteins of CIP and regulators of efflux pumps (Rehman et al., 2019; Xu et al., 2021). Furthermore, recent advances show a global response of *P. aeruginosa* to CIP stress. *P. aeruginosa* develops CIP resistance from low to high levels with distinctive proteome changes, where iron and polyamine uptake are involved in the two low levels of resistance. The MexCD-OprJ efflux pump, the *Pseudomonas* Quinolone Signal (PQS) quorum sensing, the arginine deiminase pathway, and protein degradation are characterized in the high level of resistance, while catalase, peroxidase, and DNA repair are overlapped between the low and high levels of resistance (Peng et al., 2017). Pyocin biosynthesis genes sensitize *P. aeruginosa* to CIP (Long et al., 2020). These results suggest that metabolism is related to CIP resistance in *P. aeruginosa*. It is known that antibiotic-resistant bacteria have antibiotic-resistant metabolomes, which determine the antibiotic-resistant phenotypes (Peng et al., 2015a,b; Zhang et al., 2019, 2020; Li et al., 2020; Zhao et al., 2021). However, the CIP-resistant metabolome is not defined in this pathogen.

In this study, gas chromatography–mass spectrometer (GC-MS)-based metabolomics was used to characterize metabolic profile in CIP-resistant *P. aeruginosa*. Activation of fatty acid biosynthesis was identified as one of the most characteristic features of CIP-resistant *P. aeruginosa*. Inhibition to the biosynthesis of fatty acid promotes CIP-mediated killing to lab-evolved and natural-evolved *P. aeruginosa*.

## Materials and Methods

### Bacterial Strains and Culture Conditions

*P. aeruginosa* ATCC 27853 and multidrug-resistant *P. aeruginosa* strains were obtained from our laboratory stocks. Overnight *P. aeruginosa* cultures were started in fresh Luria-Bertani (LB) medium [1% bacterial peptone, 0.5% yeast extract, and 1% sodium chloride [NaCl]] from frozen stock and incubated at 37°C. CIP-resistant *P. aeruginosa* (PA-R_CIP_) was selected from *P. aeruginosa* ATCC 27853 through sequential propagation in LB medium (1% bacterial peptone, 0.5% yeast extract, and 1% NaCl) plus 1/2 minimum inhibitory concentration (MIC) of CIP (0.125 μg/ml). Simultaneously, ATCC 27873 was sequentially generated in LB medium without antibiotic and designated CIP-sensitive *P. aeruginosa* (PA-S).

### Determination of MIC

The MIC was determined by the microdilution method, as described previously (Wiegand et al., 2008). In short, bacteria were in constant temperature shaker at 37°C at 200 rpm overnight until saturation, transferred to fresh LB liquid medium at 1:100, cultivated until the absorbance OD_600_ was 0.5, and different antibiotics were diluted by LB medium in 96-well plate for 2-fold serial dilution. An aliquot of 10 μl of 5 × 10^6^ CFU/ml logarithmic-phase bacteria was added to each well with 90 μl of a series of 2-fold dilutions of antibiotic and cultured for 16 h. The concentration observed without visible growth was the lowest antibacterial concentration, which was the MIC. At least three biological replicates were performed.

### Metabolomic Profiling

Metabolomic profiling was performed by GC-MS, as described previously (Cheng et al., 2019). In brief, overnight bacteria were reinoculated at 1:100 dilutions into 50 ml LB and cultured for 6 h until OD_600_ 1.0. Bacterial cells were harvested, washed three times with sterile saline, then collected by centrifugation at 8,000 rpm at 4°C for 5 min, and immediately quenched with liquid nitrogen. Cellular metabolites were extracted with 1 ml cold methanol containing 10 μl of 0.1 mg/ml ribitol (Sigma) as an internal standard. The cells were homogenized by ultrasound treatment (2 s, 3 s of interval, and 35% intensity) for 10 min at 4°C and then centrifuged at 12,000 rpm at 4°C for 10 min. The supernatant was transferred into a new 1.5 ml centrifuge tube. Extracts used for the GC-MS analysis were dried in a vacuum centrifuge to evaporate the methanol. Notably, 80 μl of 20 mg/ml methoxyamine hydrochloride (Sigma) in pyridine was added to the dry extracts and incubated for 180 min at 37°C. Subsequently, 80 μl of *N*-methyl-*N*-(trimethylsilyl) trifluoroacetamide (MSTFA; Sigma) was added, and the reaction was performed for 30 min at 37°C. The derivatized sample with 1 μl was injected into the DBS-MS column (Agilent Technologies). The initial temperature was 85°C for 5 min, followed by an increase to 270°C at a rate of 15°C/min, and held for 5 min. Helium was used as carrier gas at constant flow with a rate of 1 ml/min. The MS scan range was at 50–600 m/z. GC-MS data were detected with an Agilent 7890A GC equipped with an Agilent 5975C VL mass selective detector (MSD) (Agilent Technologies). Four biological repeats with two technical replicas were performed for each strain.

### Fatty Acid Metabolome Extraction for GC-MS Analysis

Sample preparation was carried out as previously described (Preez et al., 2019). In brief, the collected bacterial cells were immediately quenched with liquid nitrogen (Foshan Mulai Gas Co., Ltd., Guangdong, China) and then resuspended in 1 ml of ultrapure water (Elga LabWater, Veolia). Later, a total volume of 10 ml of a mixture of methanol and methyl tert-butyl ether (v/v, 1:1) was added. Of note, 20 μl of methyl tridecanoate was added as an analytical internal standard and mixed well. Samples were sonicated (JY92-IIN, Scientz, China) for 10 min at a 200 W power setting and centrifuged at 8,000 rpm for 5 min to transfer the supernatant to a new tube. The extract was concentrated to a dry state in a rotary vacuum centrifuge device, LABCONCO, and dissolved in 200 μl of hexane. Of note, 200 μl of a methanol solution containing 1 M KOH was added and hydrolyzed at 60°C for 30 min and then 200 μl of a methanol solution containing 14% boron trifluoride was complemented. The samples were fully derivatized at 60°C for 30 min. The mixed solution was concentrated under vacuum and dissolved in 1 ml of hexane and then 200 μl of saturated NaCl solution was added and mixed well, with anhydrous Na_2_SO_4_ for drying. An aliquot of 800 μl of the extract was transferred to a new test tube and dried in a vacuum concentrator, and the methyl-esterified product was dissolved in 100 μl of hexane and transferred to an injection tube. GC conditions were as follows: column temperature was held at 85°C for 3 min, programmed at a rate of 10°C/min to 285°C, and held for 10 min; splitless injection volume was 1 μl; total run time was 31.25 min; and the solvent delay was 5 min; and high-purity helium (99.99%) was used as carrier gas at a flow rate of 30 ml/min. MS conditions were as follows: ionization voltage was 70 eV, acquisition mass range was 50–560 amu, and scan time was 0.32 s. GC conditions were as follows: the chromatographic column temperature was 85°C, held for 3 min, programmed at a rate of 10°C/min to 285°C, and held for 10 min; non-shunt injection volume was 1 μl; the total running time was 31.25 min; the solvent delay was 5 min; and high purity helium (99.99%) was used as carrier gas with a flow rate of 30 ml/min. MS conditions were as follows: ionization voltage was 70 eV, acquisition mass range was 50–560 m/z, and scanning time was 0.32 s.

### Metabolomics Analysis

Initial chromatographic peak detection and mass spectral deconvolution were adopted by using Agilent software (Agilent version 6.0). The identification of metabolites was based on spectral matching and retention time (RT) by searching in the National Institute of Standards and Technology (NIST) Mass Spectral Library (version 2011). Data were normalized according to the peak area of internal standard (ribitol) and the total intensity. The IBM SPSS Statistics version 22 software was used to conduct significant difference analysis (non-parametric test) on the standardized data, and metabolites with *p* < 0.01 were identified as significant. Hierarchical clustering was used by R software with the package gplots (http://cran.r-project.org/web/packages/gplots/) through the distance matrix. *Z*-score was used to analyze the degree of dispersion of different metabolites after the normalized area. MetaboAnalyst version 5.0 (http://www.metaboanalyst.ca) was used to enrich the pathways of differential metabolites and metabolic pathways with *p* < 0.05 were drawn. Multivariate statistical analysis included principal component analysis (PCA), and S-plot analysis was performed by using SIMCA-P + version 12.0.1 software using Orthogonal Projections to Latent Structures Discriminant Analysis (OPLS-DA). GraphPad Prism version 8.0 was used to draw figures.

### Antibiotic Bactericidal Assay

Antibacterial assay was performed, as previously described with a few modifications (Kuang et al., 2021a). Overnight bacteria were reinoculated at 1:1,000 dilutions into 5 ml LB. Desired concentrations of antibiotics and metabolites were added and then incubated at 37°C, 200 rpm. To determine CFU per ml, 100 μl of samples were serially diluted, and an aliquot of 5 μl of each dilution was spotted in LB agar plates and cultured at 37°C for 18 h. Only the plates yielding 20–200 colonies were counted and CFU/ml was calculated. Biorepeats were run in triplicate.

### Quantitative Real-Time PCR

The bacterial cells used for qRT-PCR were the same as for antibacterial assay. qRT-PCR was performed, as described previously (Kuang et al., 2021b). Briefly, total RNA was extracted from 1 ml of a bacterial suspension at an OD_600_ of 1.0. RNA was extracted by Trizol reagent (Ambion). Reverse transcription of cDNA was performed with Evo M-MLV RT Kit with gDNA Clean for qPCR II (Accurate Biotechnology, Hunan, China). Quantitative real-time PCR (qRT-PCR) was conducted in 10 μl of the total reaction mixture in a 384-well plate, using SYBR Green Premix Pro Taq HS qPCR Kit (Accurate Biotechnology, Hunan, China), as described by the manufacturer. Cycling parameters were 95°C for 30 s, 40 cycles of 95°C for 5 s, and 60°C for 30 s. mRNA expression was detected on the LightCycler 480 system (Roche, Germany). Each sample was done with four biological repetitions with two technical replicas, and the highly conserved region of 16S rRNA was used as an internal control. Gene expression was calculated according to the 2^−Δ*ΔCT*^ method. The primers are listed in Supplementary Table 1.

### Measurement of Enzymatic Activity

Enzymatic activity of acetyl-coenzyme-A (CoA) carboxylase (ACC) was measured with a commercially available ACC activity assay kit (Solarbio Life Science, China, catalog no. BC0415). In brief, overnight saturated bacteria were transferred to fresh LB culture at 1:1,000 with or without triclosan, placed at 37°C, 200 rpm, and incubated for 6 h. Aliquots of 30 ml bacterial samples were collected. These bacteria were washed with sterile saline (0.85%) by centrifugation and then resuspended in the extract buffer, sonicated on ice (with power set to 200 W) for 5 min, and centrifuged at 4°C for 5 min at 8,000 rpm. Supernatants were transferred to a new tube, and the amount of protein was determined by the Bradford assay (Beyotime Biotechnology, Shanghai, China). According to the manufacturer's manual, 100 μg of quantified total protein was incubated at 37°C with a reaction mixture for 30 min and incubated in boiling water for 5 min. After cooling, samples were centrifuged at 10,000 *g*, 25°C for 5 min, and 20 μl of supernatant was taken with the just prepared working solution, which reacted at 37°C for 30 min. Enzymatic activities were quantitatively measured by Victor X5 multimode microplate reader at 660 nm. Results were brought into the standard curve to calculate enzyme activity. At least three biological replicates were measured for each enzyme assay.

### Measurement of Membrane Permeability

Permeability of the bacterial cell membrane was measured as previously described (Wang et al., 2014). In brief, 1 ml of bacterial cells were collected by centrifugation and resuspended in an equal volume of phosphate-buffered saline (PBS) and diluted to 10^6^ CFU/ml. Of note, 1 ml of cell suspension and 2 μl of SYTO-9 green fluorescent dye (Invitrogen, USA) were mixed together to a final concentration of 50 μM. Samples were then placed at 37°C and incubated in the dark for 15 min. All samples were immediately transferred to flow cytometry analysis tubes and detected by flow cytometer to obtain green fluorescence intensity. The intensity of the green fluorescence signal is related to the permeability of the cell membrane. The high value of fluorescence intensity means high permeability. At least three independent biological replicates were performed for each sample.

### Measurement of Intracellular CIP

Overnight saturated *P. aeruginosa* CIP-resistant bacteria were transferred to fresh LB medium at a ratio of 1:1,000, which contained 2 μg/ml CIP with or without 1 μg/ml triclosan and incubated at 37°C with 200 rpm for 6 h. An aliquot of 30 ml bacterial solution was collected and washed three times with 0.85% sterile saline. After centrifugation (8,000 rpm, 5 min), 600 μl of PBS was added for ultrasonic cell disruption (2 s on/3 s off, with an intensity of 35% for 10 min). An aliquot of 100 μl of supernatant was taken and added to *Escherichia coli* with OD_600_ = 0.2 (resuspended in M9 medium) and incubated at 37°C with 200 rpm for 6 h. After a series of dilutions, 5 μl was dropped onto an LB square plate containing 2% agar and incubated at 37°C for 18 h. The number of colonies on the plate was counted, and the survival rate was calculated.

## Results

### Metabolic Profiles of PA-R

**Figure 1:**
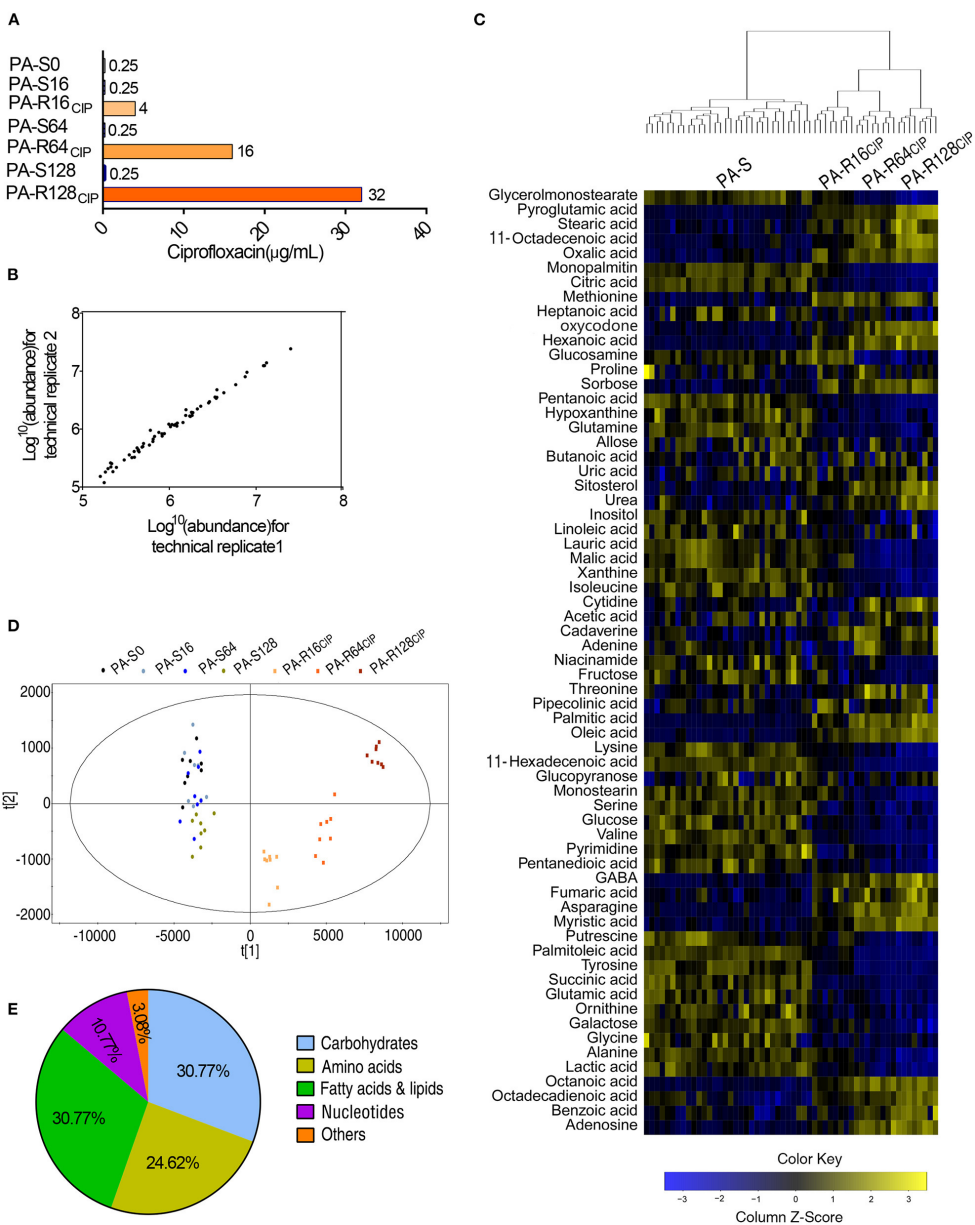
Minimum inhibitory concentration (MIC) and metabolic profiles of PA-S and three PA-R strains. **(A)** MIC of seven *P. aeruginosa* strains to ciprofloxacin. **(B)** Correlation coefficient of two technical replications. The Pearson correlation coefficient between technical replicates varies between 0.996 and 0.999. **(C)** Heat map of the differential abundance of metabolites (row). Blue and yellow indicated a decrease and increase of the metabolites scaled to mean and standard deviation of row metabolite level, respectively (refer to color scale). **(D)** Scores plot of OPLS-DA model between PA-S and PA-R. Each dot represented the technical replicate analysis of samples in the plot. **(E)** Percentage of metabolites in every category. Sixty-five metabolites were searched against KEGG for their categories.

To understand the global metabolic change of CIP-resistant *P. aeruginosa, P. aeruginosa* ATCC 27853 was sequentially cultured in a medium with and without 1/2 MIC of CIP. This led to CIP-resistant *P. aeruginosa* with 16-, 64-, and 128-fold MICs (PA-R16CIP, PA-R64CIP, and PA-R128CIP, respectively) and their corresponding sequential generations without CIP (PA-S16, PA-S64, and PA-S128, respectively; their MIC was 0.25 μg/ml), compared to CIP-sensitive parental strain (PA-S0, MIC, 0.25 μg/ml) (Figure 1A). These strains were used for GC-MS analysis. Four biological replicates with two technical replicas were performed in each strain, yielding 56 data sets. GC-MS fingerprint was obtained, and the Agilent software was used for data integration, RT correction, and metabolite identification. A total of 65 metabolites were determined with reliable signals. The Spearman correlation coefficient was calculated between two technical repeats for technical repeatability according to Correl's calculation formula. The correlation coefficient was obtained between 0.996 and 0.999, guaranteeing repeatability (Figure 1B). The peak area of the 65 metabolites was calibrated by ribitol and then the total amount of the metabolites. The resulting heat map was shown in Figure 1C. The seven strains were clustered based on sensitivity and resistance to CIP. The sensitivity and resistance groups include PA-S0, PA-S16, PA-S64, PA-S128 (the former three together PA-S) and PA-R16CIP, PA-R64CIP, and PA-R128CIP (together PA-R). The three PA-R strains were further subclustered, while the four PA-S strains were not separated. These results indicate that the metabolomes changed with the development of different MICs of resistance, while PA-S did not cause the metabolic difference observed for PA-R. PCA of these 65 metabolites showed that factor t[1] distinguished PA-S from PA-R, while factor t[2] separated the deviation of PA-S, PA-R16CIP, and PA-R64CIP from PA-R128CIP (Figure 1D). The 65 metabolites were divided into five categories. Specifically, 30.77, 24.62, 30.77, and 10.77% belonged to carbohydrate, amino acid, fatty acid and lipid, and nucleotide, respectively (Figure 1E). Therefore, metabolic states are altered in these PA-R strains.

### Differential Metabolic Profiles of PA-R

**Figure 2:**
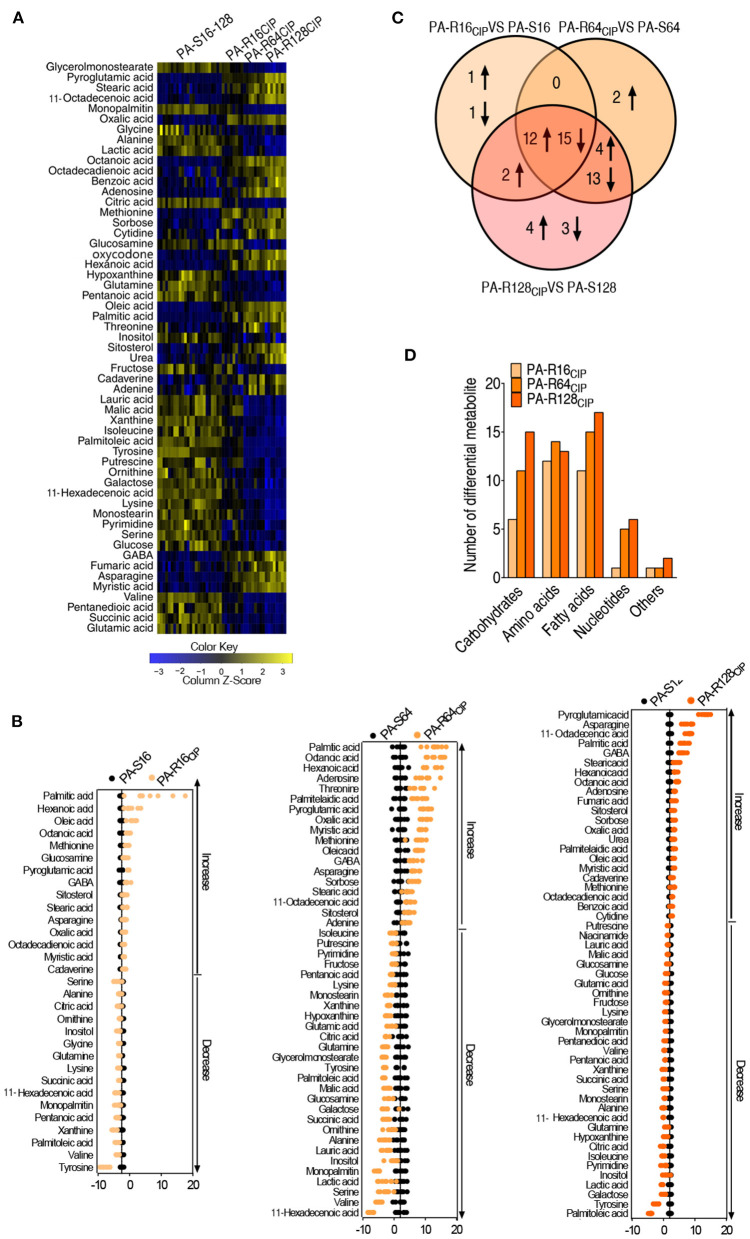
Analysis of differential abundance metabolites of *P. aeruginosa*. **(A)** Differential metabolic profiles in PA-R16_CIP_, PA-R64_CIP_, and PA-R128_CIP_ were compared with PA-S16, PA-S64, and PA-S128 mixed together. **(B)** Z-score plots. PA-R16_CIP_, PA-R64_CIP_, and PA-R128_CIP_ were compared with PA-S16, PA-S64, and PA-S128, respectively. **(C)** Venn diagram of the total differential metabolites between PA-R16_CIP_, PA-R64_CIP_, and PA-R128_CIP_ with their control group. **(D)** The number of differential abundant metabolites from PA-R16_CIP_, PA-R64_CIP_, and PA-R128_CIP_ compared with their control group in every category.

Out of 65 metabolites, 55 metabolites had differential abundances by using non-parametric analysis with *p* < 0.01, as shown in Figure 2A. Interestingly, most of the differential abundance of metabolites displayed a gradient change with the increased CIP resistance (Figure 2A). Deviation of the differential abundance of metabolites was exhibited by comparison between PA-R and their corresponding PA-S (PA-R16_CIP_ vs. PA-S16, PA-R64_CIP_ vs. PA-S64, and PA-R128_CIP_ vs. PA-S128) using *Z*-value. The larger deviation was detected in upregulated than downregulated metabolites. The first four largest upregulated metabolites included palmitic acid, hexanoic acid, oleic acid, and octanoic acid in PA-R16_CIP_, palmitic acid, octanoic acid, hexanoic acid, and adenosine in PA-R64_CIP_, and pyroglutamic acid, asparagine, octadecenoic acid, and palmitic acid in PA-R128_CIP_. Among them, palmitic acid, oleic acid, and octadecenoic acid work for the biosynthesis of fatty acids (Figure 2B). Among these differential metabolites, 27 metabolites were overlapped; 17, 2, and 0 were shared between PA-R64_CIP_ and PA-R128_CIP_, PA-R16_CIP_ and PA-R128_CIP_, and PA-R16_CIP_ and PA-R64_CIP_, respectively. In addition, 2, 2, and 7 were detected only in PA-R16_CIP_, PA-R64_CIP_, and PA-R128_CIP_, respectively (Figure 2C). A number of the differential abundance of metabolites were exhibited according to kyoto encyclopedia of genes and genomes (KEGG) metabolic categories. The numbers rose with the MIC except for amino acids (Figure 2D). These results indicate that the shifted metabolomes are related to MIC.

### Metabolic Pathway Enrichment of PA-R

**Figure 3:**
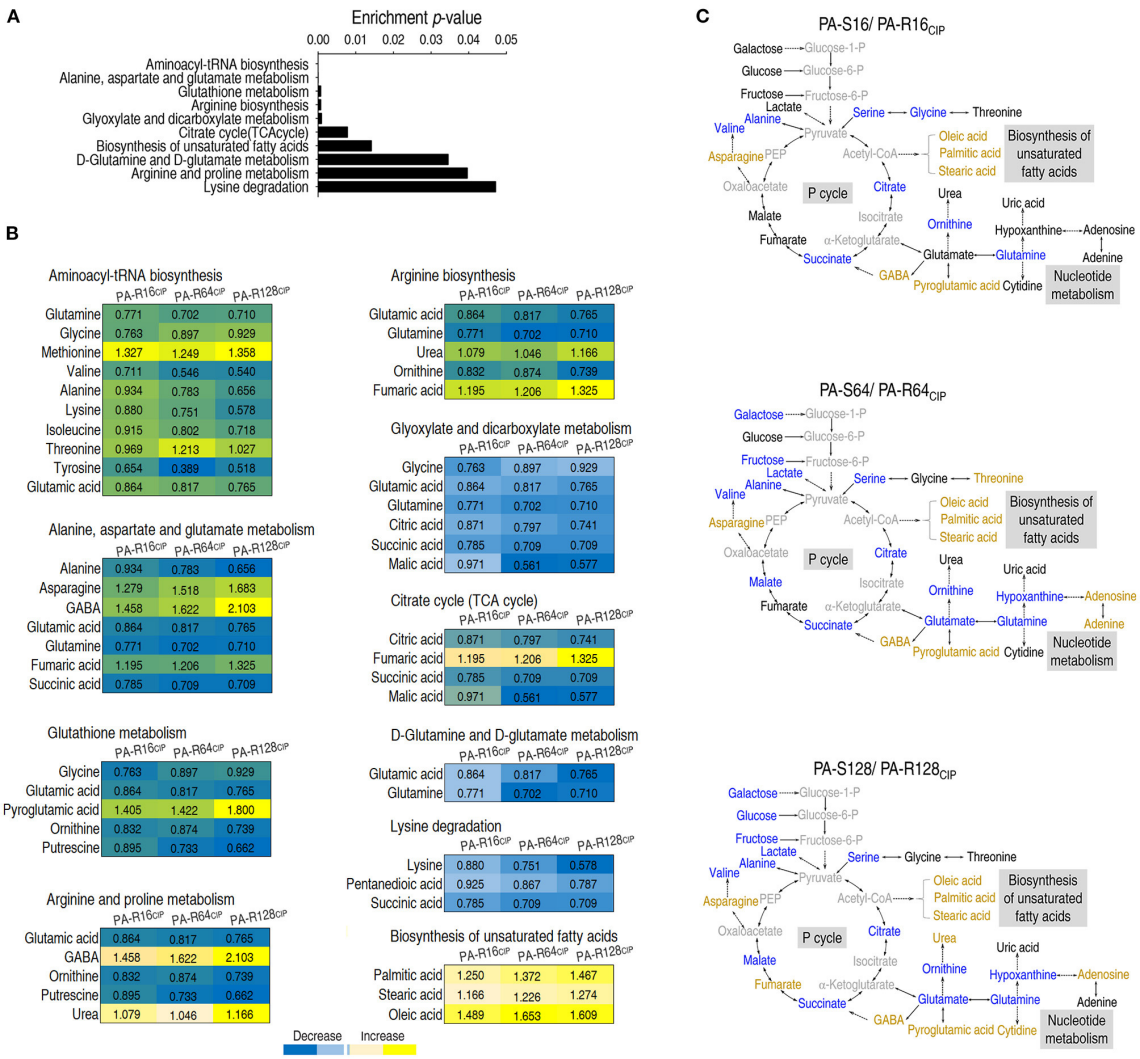
Analysis of pathway enrichment in ciprofloxacin-resistant *P. aeruginosa*. **(A)** Pathway enrichment of differential abundant metabolites. **(B)** Integrative analysis of differential abundant metabolites in enriched pathways. Blue and yellow represented a decrease and increase in the abundance of metabolites, respectively. **(C)** MetaMapp visualization of metabolomic data highlighting the differential metabolic regulation from PA-R16_CIP_, PA-R64_CIP_, and PA-R128_CIP_ was compared with PA-S16, PA-S64, and PA-S128, respectively. Yellow, increased abundance; Blue, decreased abundance; Black, no difference; Weak black, not detected.

All metabolites are chemically transformed in reactions that belong to pathways, essential for the correct functioning of a biological system. Metabolic pathway enrichment analysis can discover key metabolic pathways and provides a comprehensive view of differential abundances of metabolites belong to. Metabolic pathway enrichment was performed through the online website MetaboAnalyst (https://www.metaboanalyst.ca/) data analysis. A total of 10 metabolic pathways were enriched (*p* < 0.05). The order on *p*-value ranked from low to high as follows: aminoacyl-tRNA biosynthesis, alanine, aspartate and glutamate metabolism, glutathione metabolism, arginine biosynthesis, glyoxylate and dicarboxylate metabolism, citrate cycle [the tricarboxylic acid (TCA) cycle], biosynthesis of unsaturated fatty acids, D-glutamine and D-glutamate metabolism, arginine and proline metabolism, and lysine degradation (Figure 3A). Among them, all metabolites detected were elevated only in the biosynthesis of unsaturated fatty acids, while all or most metabolites detected were reduced in other metabolic pathways (Figure 3B). An overview of the metabolic changes was shown in PA-R16_CIP_, PA-R64_CIP_, and PA-R128_CIP_ (Figure 3C). These results indicate that the enhanced biosynthesis of unsaturated fatty acids can be a clue to explore the mechanism of CIP resistance.

### Identification of Biomarkers of PA-R

**Figure 4:**
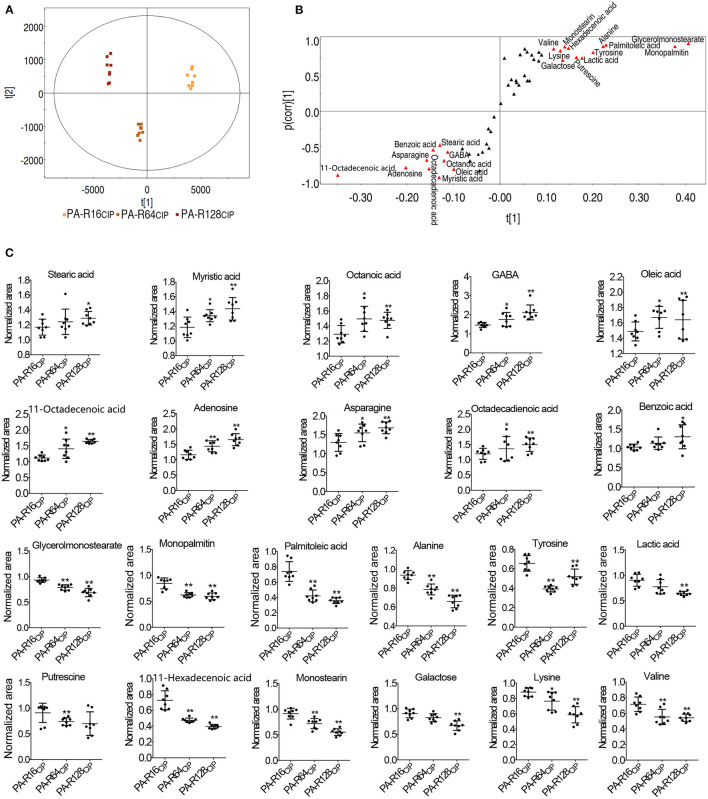
Analysis of potential biomarkers of ciprofloxacin-resistant *P. aeruginosa*. **(A)** Principal component analysis of *P. aeruginosa*. **(B)** S-plot generated from OPLS-DA. Triangle represents individual metabolite, where potential biomarkers are highlighted with red, which was greater or equal to 0.05 and 0.5 for absolute value of covariance *p* and correlation *p* (corr), respectively. **(C)** Scatter plot of key metabolite abundance. Results are displayed as mean ± SEM, and significant differences are identified (*p < 0.05, **p < 0.01) as determined by two-tailed Student's *t*-test.

Differential abundances of metabolites were analyzed by PCA and S-plot for the identification of biomarkers. PCA analysis showed that PA-R16_CIP_, PA-R64_CIP_, and PA-R128_CIP_ were separately located in different quadrates (Figure 4A). Then, S-plot was used to identify metabolites with absolute values of the S-plot variation weight *t* and correlation coefficient *p* (corr) >0.05 and 0.5, respectively, as biomarkers. This led to the identification of 22 biomarkers, which were marked with red triangles (Figure 4B). Among them, 10 metabolites (i.e., stearic acid, myristic acid, octanoic acid, γ-aminobutyric acid (GABA), oleic acid, octadecenoic acid, adenosine, asparagine, octadecadienoic acid, and benzoic acid) and 12 metabolites (i.e., glycerol monostearate, monopalmitin, palmitoleic acid, alanine, lactic acid, tyrosine, putrescine, hexadecenoic acid, monostearin, galactose, lysine, and valine) were elevated and decreased with increasing MIC values, respectively (Figure 4C). Interestingly, stearic acid, myristic acid, oleic acid, octadecenoic acid, octadecadienoic acid, glycerol monostearate, monopalmitin, palmitoleic acid, hexadecenoic acid, and monostearin represent metabolites that belong to the biosynthesis of fatty acids. These results indicate that the biosynthesis of fatty acids may play a role in PA-R strains.

### Activation of Fatty Acid Biosynthesis and Repression of Fatty Acid Degradation

**Figure 5:**
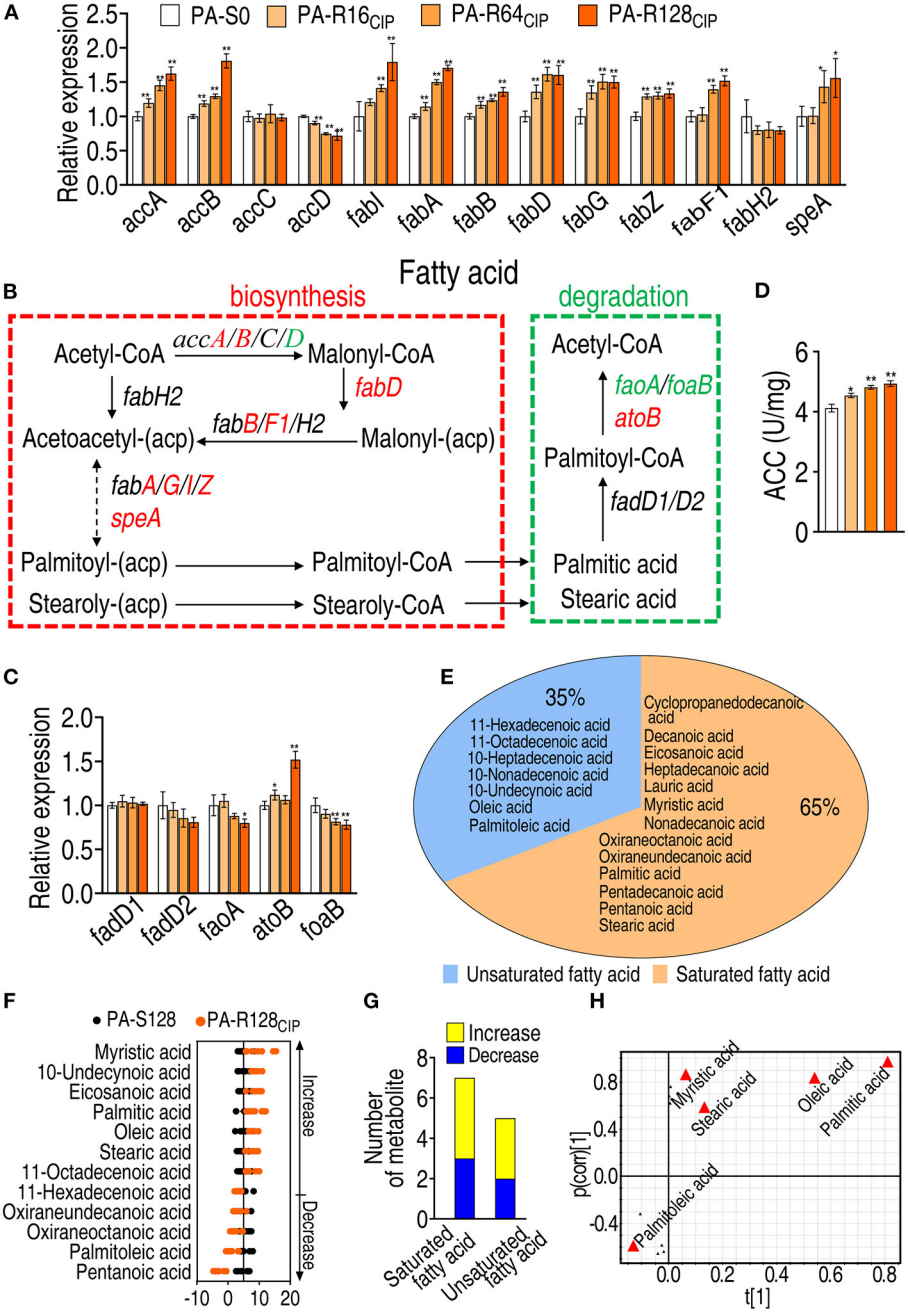
Analysis of fatty acid biosynthesis and degradation in AP-R_CIP_. **(A)** qRT-PCR for the expression of genes encoding fatty acid biosynthesis in PA-S0, PA-R16_CIP_, PA-R64_CIP_, and PA-R128_CIP_. **(B)** Outline for expression of genes in fatty acid metabolism pathway. Red: increase, green: decrease. **(C)** qRT-PCR for the expression of genes encoding fatty acid degradation in PA-R16_CIP_, PA-R64_CIP_, and PA-R128_CIP_. **(D)** Activity of acetyl-coenzyme-A (CoA) carboxylase (ACC) in PA-R16_CIP_, PA-R64_CIP_, and PA-R128_CIP_. **(E)** Profile of fatty acid metabolome in PA-R128_CIP_. **(F)** Z-score plot of differential fatty acids in PA-R128_CIP_. **(G)** Number of differential saturated and unsaturated fatty acids in PA-R128_CIP_. **(H)** S-plot generated from OPLS-DA based on the differential fatty acids in data **(F)**. Triangle represents individual metabolite, where potential biomarkers are highlighted with red, which is greater or equal to 0.05 and 0.5 for the absolute value of covariance *p* and correlation *p* (corr), respectively. Results are displayed as mean ± SEM and at least three biological repeats are performed. Significant differences are identified *p < 0.05, **p < 0.01.

To further demonstrate the elevated fatty acid biosynthesis and explore why fatty acids are increased, qRT-PCR was used to measure the expression of genes encoding fatty acid biosynthesis and fatty acid degradation. The expression of the 10 genes was elevated, 1 reduced, and 2 remained unchanged in fatty acid biosynthesis (Figures 5A,B). The expression of the 1 gene was elevated, 2 reduced, and 2 remained unchanged in fatty acid degradation (Figures 5B,C). ACC works for the first and rate-limiting step of *de novo* fatty acid biosynthesis. Consistently, the activity of ACC was elevated (Figure 5D). These results validate the activation of fatty acid biosynthesis in PA-R strains.

Moreover, fatty acid metabolome was detected in PA-S128 and PA-R128_CIP_ by GC-MS. A total of 20 fatty acids were identified, with 13 saturated fatty acids and seven unsaturated fatty acids (Figure 5E). Deviation of 12 differential abundances of fatty acids was exhibited using the *Z*-value. Among them, seven were upregulated and five were downregulated (Figure 5F). The seven upregulated fatty acids consisted of four saturated fatty acids (i.e., myristic acid, stearic acid, palmitic acid, and eicosanoic acid) and three unsaturated fatty acids (i.e., oleic acid, 11-octadecanoic acid, and 10-undecynoic acid), while the five downregulated fatty acids included three saturated fatty acids (i.e., oxiraneundecanoic acid, oxiraneoctanoic acid, and pentanoic acid) and two unsaturated fatty acids (i.e., palmitoleic acid and 11-hexadecenoic acid) (Figure 5G). S-plot identified 3 upregulated saturated fatty acids (i.e., myristic acid, stearic acid, and palmitic acid) and one upregulated (i.e., oleic acid) and one downregulated unsaturated fatty acid (i.e., palmitoleic acid) as biomarkers (Figure 5H). These results indicate that upregulated fatty acids are dominant in the altered fatty acid metabolome.

### Activation of Fatty Acid Biosynthesis Contributes to CIP Resistance

**Figure 6:**
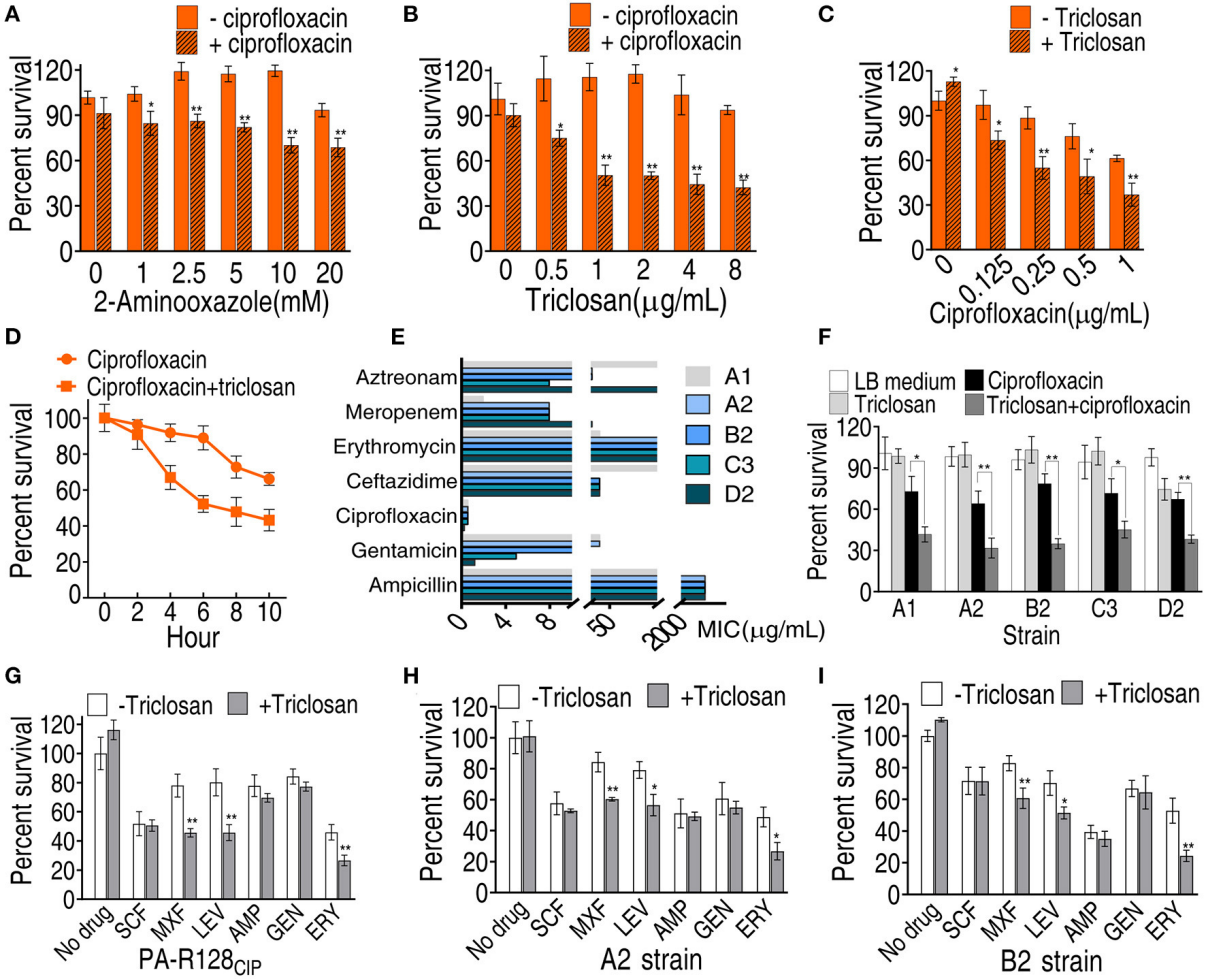
Viability of PA-R128_CIP_ and clinically multidrug-resistant *P. aeruginosa* in the presence of antibiotics or/and triclosan. **(A,B)** Percent survival of PA-R128_CIP_ in the indicated concentrations of aminooxazole or triclosan plus ciprofloxacin (0.25 μg/ml). **(C)** Percent survival of PA-R128_CIP_ in the indicated concentrations of ciprofloxacin plus triclosan (1 μg/ml). **(D)** Percent survival of PA-R128_CIP_ in the indicated incubation periods and in the presence of ciprofloxacin (0.25 μg/ml) plus triclosan (1 μg/ml). **(E)** MIC of clinical *P. aeruginosa*. **(F)** Percent survival of clinically multidrug-resistant *P. aeruginosa* in the presence of ciprofloxacin (0.025 μg/ml) or/and triclosan (1μg/ml). **(G–I)** Percent survival of PA-R128_CIP_ **(G)**, A2 **(H)**, and B2 **(I)** in the presence of different antibiotics or/and triclosan (1 μg/ml). Results are displayed as mean ± SEM and three biological repeats are performed. Significant differences are identified *p < 0.05, **p < 0.01.

To know whether the activated fatty acid biosynthesis is related to CIP resistance, inhibitors 2-aminooxazole and triclosan were used to block the biosynthesis. 2-Aminooxazole inhibits ACC to reduce acetyl-CoA into the biosynthesis of fatty acids (Polyak et al., 2012). Triclosan is a potent inhibitor of FabI to reduce acyl carrier protein (ACP) into the biosynthesis of saturated fatty acids (Heath et al., 1998; Tkachenko et al., 2007). 2-Aminooxazole and triclosan elevated CIP-mediated killing in a dose-dependent manner (Figures 6A,B). However, stronger inhibition was detected in triclosan than 2-aminooxazole and, thereby, only triclosan was used in the following study. The triclosan-mediated killing was also increased with increasing doses of CIP and incubation periods (Figures 6C,D). To test the feasibility of the triclosan-potentiated killing by CIP, five clinically multidrug-resistant *P. aeruginosa* strains were used. A1 strains were not sensitive to gentamicin, CIP, ceftazidime, and aztreonam. A2 and B2 strains were not sensitive to gentamicin, CIP, ceftazidime, meropenem, and aztreonam. C3 strains were not sensitive to gentamicin, CIP, ceftazidime, and meropenem. D2 strains were not sensitive to ceftazidime, meropenem, and aztreonam (Figure 6E). The lower viability of these strains was detected in the presence of triclosan (Figure 6F). Further experiments showed that triclosan also potentiated other fluoroquinolones [LEV and moxifloxacin [MXF]] and erythromycin to effectively kill PA-R128_CIP_, A2, and B2 strains, but not cefoperazone/sulbactam, ampicillin, and gentamicin (Figures 6G–I). MexCD-OprJ is the main CIP efflux pump and possibly uses long-chain fatty acids as substrates (Stickland et al., 2010). Thus, *mexCD-oprJ* gene expression was quantified. No difference in *mexCD-oprJ* expression was measured between PA-S and PA-R strains, and the expression was also not affected by triclosan (Supplementary Figure 1). These results support the conclusion that elevated fatty acid biosynthesis contributes to fluoroquinolone resistance.

### Triclosan Inhibits the Biosynthesis of Fatty Acids

To validate the above results, the effect of triclosan on the biosynthesis of fatty acids is examined. qRT-PCR analysis was used to quantify the 13 and five genes encoding biosynthesis and degradation of fatty acids, respectively, in the three PA-R strains with or without triclosan. Among the 13 genes, nine genes exhibited reduced expression when triclosan was added (Supplementary Figure 2A). These data were interesting because triclosan only targets *fabI*, suggesting that triclosan-mediated inhibition affects other gene expressions of the same pathway. The lower activity of ACC of the three PA-R strains was quantified in a medium containing triclosan (Supplementary Figure 2B). In contrast, triclosan elevated the expression of *atoB* but did not affect the expression of the other four genes in the degradation of fatty acids (Supplementary Figure 2C). Consistently, triclosan inhibited the expression of the nine same genes and activity of ACC (Supplementary Figures 2D,E), whereas the inhibitor did not affect the expression of genes encoding degradation of fatty acids except for the elevated expression of *atoB* in B2 strain (Supplementary Figure 2F). These results support the conclusion that the triclosan-potentiated CIP-mediated killing is attributed to the reduced biosynthesis of fatty acids.

**Figure 7:**
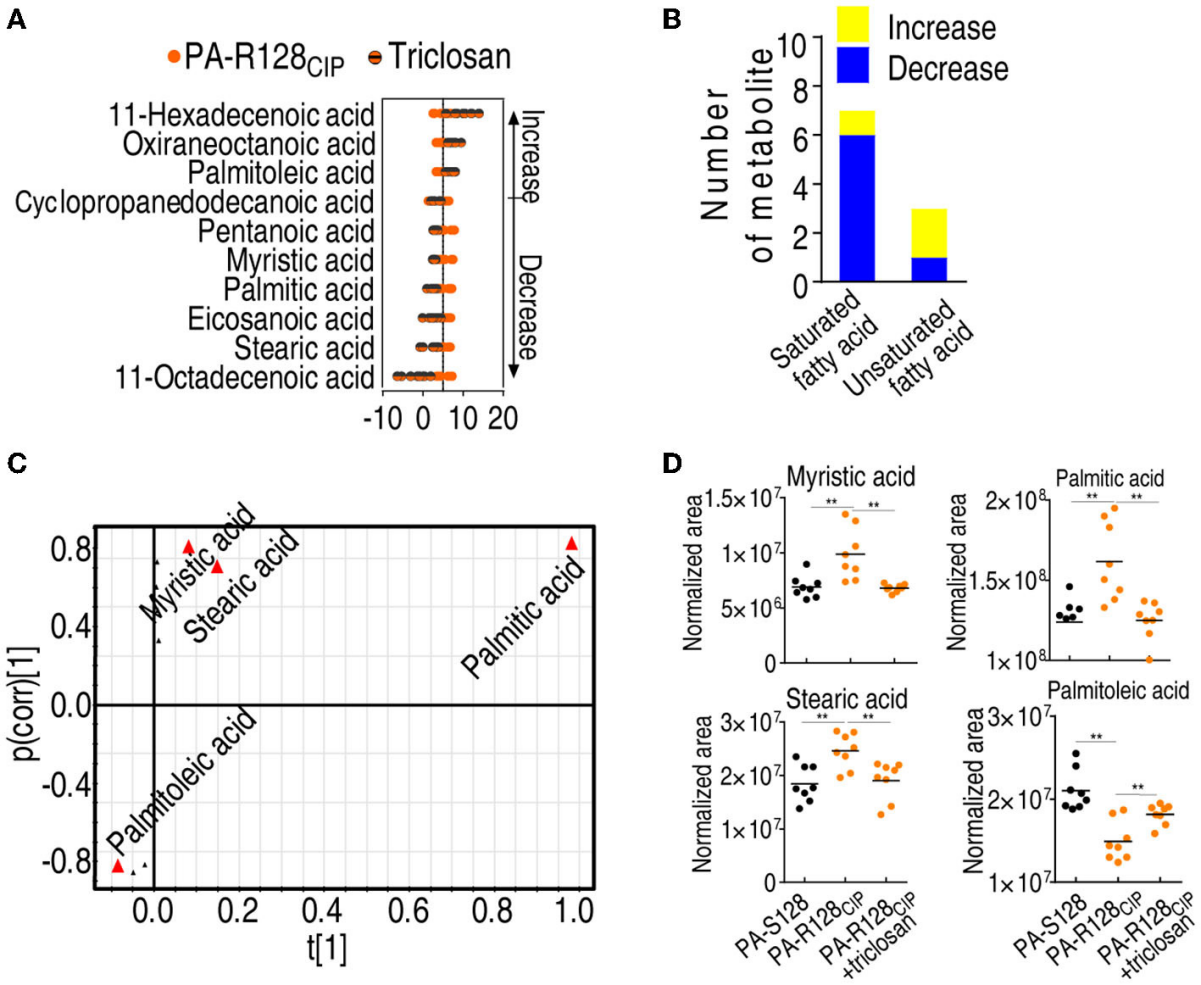
The effect of triclosan on fatty acid metabolism. **(A)** Z-score plot of differential fatty acids in the presence of triclosan (1 μg/ml) in PA-R128_CIP_. **(B)** Number of differential saturated and unsaturated fatty acids between PA-R128_CIP_ and PA-R128_CIP_ plus triclosan (1 μg/ml). **(C)** S-plot generated from OPLS-DA based on the differential fatty acids in data **(A)**. Triangle represents individual metabolite, where potential biomarkers are highlighted with red, which is greater or equal to 0.05 and 0.5 for absolute value of covariance *p* and correlation *p* (corr), respectively. **(D)** Comparison for differential abundance of fatty acids in data **(C)**. Results are displayed as mean ± SEM and three biological repeats are performed. Significant differences are identified *p < 0.05, **p < 0.01.

Fatty acid-based metabolomics was also used to investigate the effect of triclosan on the metabolome. When triclosan was used in PA-R128_CIP_, three and seven fatty acids were upregulated and downregulated, respectively (Figure 7A). The inhibitor upregulated one saturated fatty acid and two unsaturated fatty acids and downregulated 6 saturated fatty acids and 1 unsaturated fatty acid (Figure 7B). Among them, myristic acid, stearic acid, palmitic acid, and palmitoleic acid were identified as the crucial biomarkers by S-plot (Figure 7C). Triclosan upregulated palmitoleic acid and downregulated palmitic acid, myristic acid, and stearic acid to be normal (Figure 7D). These findings are consistent with the association of the triclosan-potentiated CIP-mediated killing with the biosynthesis of fatty acids.

### Cell Membrane Permeability Contributes to Fatty Acid Biosynthesis-Related Resistance

**Figure 8:**
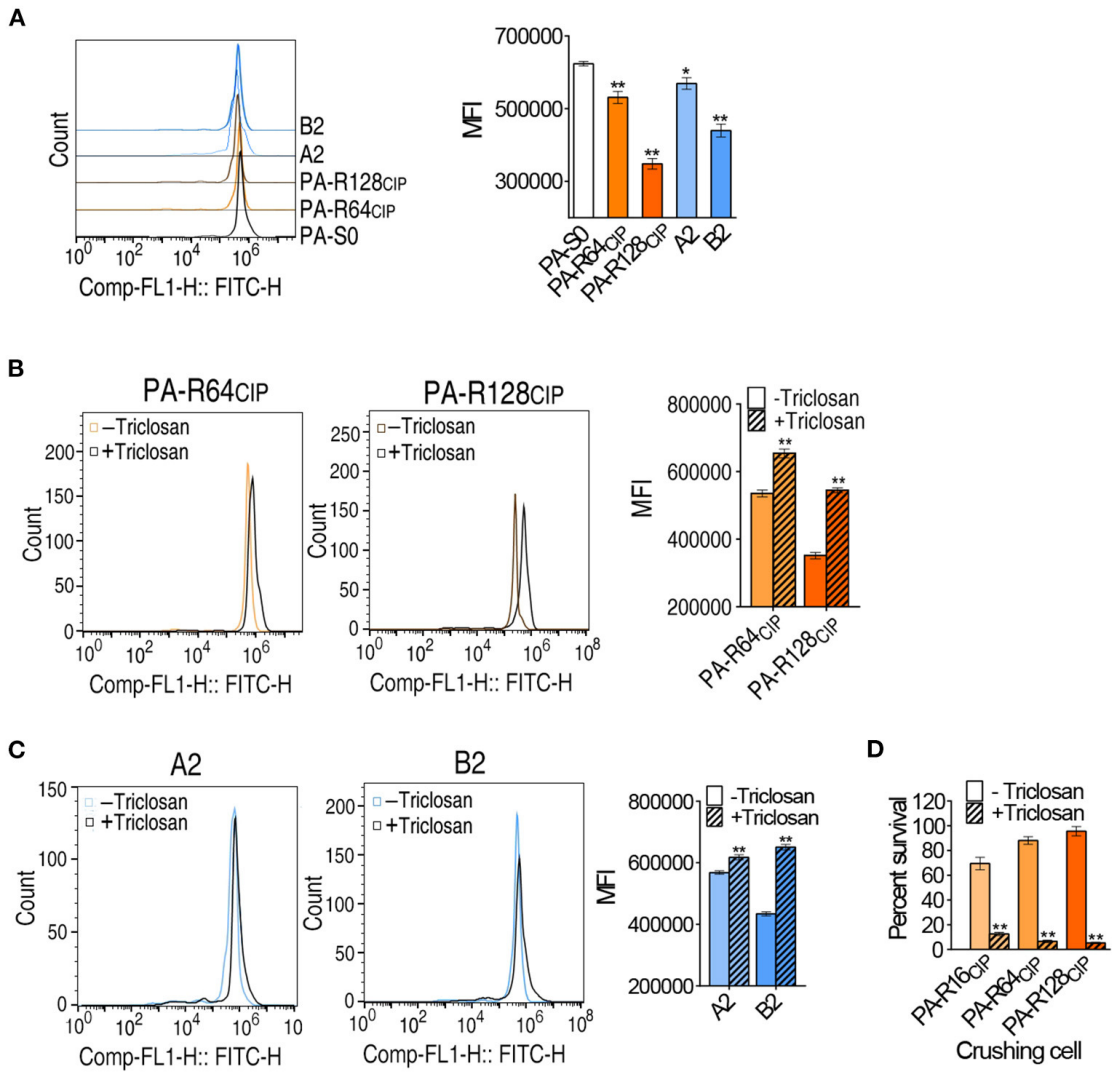
Membrane permeability among *P. aeruginosa* and survival rate of lysate against *E. coli*. **(A)** Green fluorescence signal intensity and single parameter histogram among *P. aeruginosa*. **(B,C)** Green fluorescence signal intensity in the presence of triclosan and single parameter histogram. The left shift of fluorescence peak diagram indicates the decrease of membrane permeability, the right shift of peak diagram indicates the increase of membrane permeability, and the MFI indicates the fluorescence signal intensity. **(D)** Effects of *P. aeruginosa* lysate on *E. coli* K12BW25113 survival. Results are shown as mean ± SEM and at least three biological repeats are performed. Significant differences are identified *p < 0.05, **p < 0.01.

To understand the enhanced biosynthesis of fatty acids responsible for CIP resistance, membrane permeability was measured since the incorporation of fatty acids variably affects membrane permeability (Royce et al., 2013). Membrane permeability was lower in PA-R64_CIP_ and PA-R128_CIP_ than PA-S0. In addition, lower membrane permeability was measured in clinically multidrug-resistant strains A2 and B2, which was similar to or lower than PA-R64_CIP_ (Figure 8A). When the inhibitor triclosan was added, membrane permeability was elevated in PA-R64_CIP_ and PA-R128_CIP_ (Figure 8B). Similarly, triclosan also led to elevated membrane permeability in clinically multidrug-resistant strains A2 and B2 (Figure 8C). Logically, the elevated membrane permeability should promote intracellular CIP concentration. To demonstrate this, cells incubated with CIP with or without triclosan were crushed in saline solution. The saline solution from crushed cells was used to test antibacterial capability. Viability was lower in cells containing triclosan, suggesting more CIP in cells containing triclosan (Figure 8D). These results indicate that the enhanced biosynthesis of fatty acids elevates membrane permeability and inhibits CIP uptake.

## Discussion

It is suggested that the metabolic flexibility of *P. aeruginosa* could lead to new strategies to combat bacterial infection (Mielko et al., 2019; Allobawi et al., 2020; Stephen, 2020; Kuang et al., 2021a; Moyne et al., 2021). This study adopts GC-MS-based metabolomics to explore metabolic profiles of PA-R_CIP_ compared to PA-S. The comparison is performed among seven strains, namely, PA-S16, PA-S64, PA-S128, PA-R16_CIP_, PA-R64_CIP_, PA-R128_CIP_, and their parent strain PA-S0. Cluster analysis shows that four controls, namely, PA-S0, PA-S16, PA-S64, and PA-S128 are grouped together, whereas PA-R16_CIP_, PA-R64_CIP_, and PA-R128_CIP_ are separated from PA-S and further subclustered each other. Twenty-two biomarkers are identified, almost of which changed with MIC gradient. These results support the conclusion that antibiotic-sensitive and antibiotic-resistant bacteria have antibiotic-sensitive and antibiotic-resistant metabolomes, respectively (Peng et al., 2015a,b; Zhao et al., 2021).

The phenotypic evolution of *P. aeruginosa* population changes occurs in the presence of subinhibitory concentrations of CIP (Wassermann et al., 2016; Ahmed et al., 2018), which can be used to predict the *in vivo* evolutionary trajectories (Ahmed et al., 2020). Therefore, this study uses the subinhibitory concentrations of CIP for the selection of CIP-resistant *P. aeruginosa*. Different from most reports on comparative metabolomics (Jiang et al., 2020; Wang et al., 2020; Kuang et al., 2021a,b), this study uses paired samples to understand metabolic states with MIC-gradient changes and explores the CIP-resistant metabolic mechanisms. The CIP-resistant metabolome of *P. aeruginosa* characterizes the elevated biosynthesis of fatty acids, which is the most characteristic feature since all metabolites detected are elevated only in the biosynthesis of fatty acids out of 10 enriched metabolic pathways. The elevation is further validated by qRT-PCR, enzyme activity measurement, and fatty acid metabolome. Notably, ACC catalysis is the first committed step in fatty acid biosynthesis. The enzyme has four subunits, encoded separately by *accA, accB, accC*, and *accD*. This study shows that the expressions of *accA* and *accB* were elevated, *accD* was reduced, and *accC* remained unchanged in PA-R, but the activity of ACC is elevated. Upregulated and downregulated expressions of genes encoding an enzyme with multisubunit have been reported in response to antibiotic stress (Chen et al., 2016; Zhang et al., 2020). Consistently, *accA* plays a major role in fatty acid synthesis (Kim, 1997). Furthermore, among the 22 biomarkers identified by S-plot, 10 were located in the biosynthesis of fatty acids. These together support the conclusion that the enhanced biosynthesis of fatty acids is related to CIP resistance.

Enhanced biosynthesis of fatty acids is associated with the acquisition of quinolone antibiotic resistance that has been revealed in bacteria, including *Vibrio alginolyticus, Edwardsiella tarda*, and *Xanthomonas oryzae* (Cheng et al., 2018; Su et al., 2021; Wang et al., 2021). In addition, RNA-Seq analysis of *P. aeruginosa* after treatment with a subinhibitory CIP concentration identifies 15 KEGG-enriched pathways, where biosynthesis of fatty acids and metabolism of fatty acids are included (Molina-Mora et al., 2020). The total cellular protein contents are decreased after sub-MIC of CIP treatment in company with the increase of total lipids and phospholipids and the decrease of neutral lipids in *P. aeruginosa* (Yehia et al., 2015). The altered fatty acid and triacylglycerol metabolism are detected in tuberculosis after being exposed to CIP (Knoll et al., 2021). Inhibition of fatty acid biosynthesis promotes norfloxacin-mediated killing to methicillin-resistant *Staphylococcus aureus* (MRSA) (Sinha et al., 2019). However, the role of fatty acid biosynthesis in quinolone antibiotic resistance is largely unknown in *P. aeruginosa*. This study demonstrates the activation of fatty acid biosynthesis at the levels of gene expression, enzyme activity, and metabolites in CIP-resistant *P. aeruginosa*. When the biosynthesis is inhibited by triclosan, which is confirmed by reduced gene expression, enzyme activity, and fatty acid metabolome, CIP-mediated killing is promoted. These findings are derived from understanding that bacterial metabolic environment contributes to bacterial sensitivity to antibiotics, and antibiotic-resistant metabolome can be reverted to antibiotic-sensitive metabolome using metabolome-reprogramming (Peng et al., 2015a; Su et al., 2018; Zhang et al., 2019, 2020; Li et al., 2020; Zhao et al., 2021). Our very recent report indicates that inactivation of the pyruvate cycle and nitric oxide (NO) biosynthesis is identified as characteristic features of cefoperazone-sulbactam resistance in naturally and artificially evolved *P. aeruginosa* strains with cefoperazone-sulbactam resistance, which can be reverted by exogenous nitrite and nitrate (Kuang et al., 2021a). Therefore, a comprehensive analysis of the antibiotic-resistant metabolic states will be helpful in understanding the antibiotic-resistant mechanisms, identifying crucial biomarkers to reprogram the antibiotic-resistant metabolic state to antibiotic-sensitive metabolic state, thereby potentiating conventional antibiotics to control antibiotic-resistant bacteria.

The content and ratio of fatty acids affect membrane permeability (Royce et al., 2013), which is related to antibiotic uptake. In contrast, the anti-bacterial effect of fluoroquinolones is related to efficient cellular membrane penetration, where CIP translocation crosses a lipid bilayer composed of unsaturated phosphatidylcholine molecules (Cramariuc et al., 2012). Consistently, this study shows that CIP resistance causes the alteration of the fatty acid metabolome, leading to higher saturated fatty acids and lower unsaturated fatty acids. Replacement of saturated with unsaturated fatty acids is linked with the reduced membrane permeability and CIP uptake in PA-R strains and clinically multidrug-resistant strains. Triclosan can deplete the membrane potential in *P. aeruginosa* biofilms inhibiting aminoglycoside-induced adaptive resistance (Maiden and Waters, 2020), but we found that triclosan promotes the alteration of the fatty acid metabolome of PA-R to return to that of PA-S and, thereby, elevates CIP-mediated killing by increasing membrane permeability and CIP uptake; this discrepancy may suggest that triclosan regulates antibiotic effect through different mechanisms. Our results suggest that the biosynthesis of fatty acids-related CIP resistance is mostly attributed to membrane permeability.

## Conclusion

**Figure 9:**
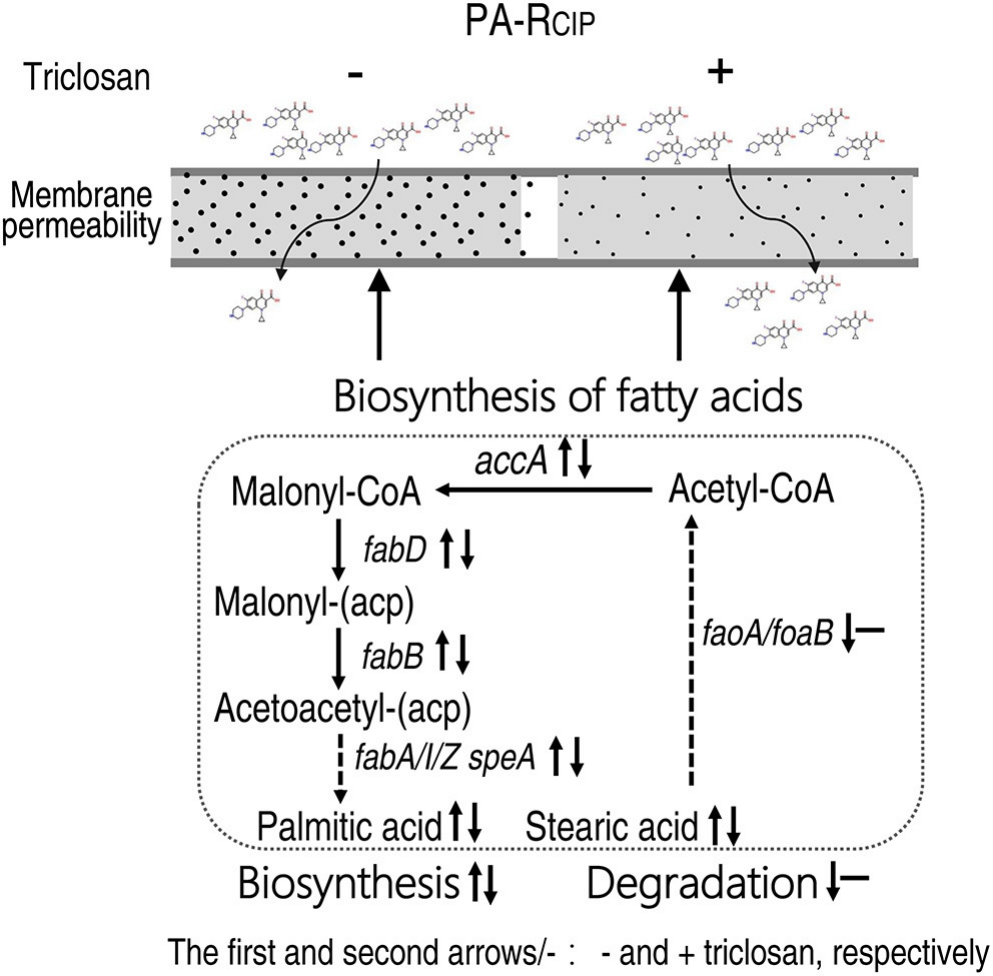
Model showing the proposed metabolic regulation for ciprofloxacin resistance.

This study adopts GC-MS-based metabolomics to explore CIP metabolic resistance mechanisms. The MIC-gradient metabolomes show that the biosynthesis of fatty acid is the only elevated metabolic pathway with increasing MIC and, thereby, it is used as a key clue to understanding the resistance mechanism. qRT-PCR, enzyme measurement, and fatty acid metabolome confirm the elevation. Functional experiments with the pathway inhibitor triclosan demonstrate that CIP-mediated killing is promoted together with reduced gene expression and enzyme activity and the recovered fatty acid metabolome. The promotion is effective for both lab-evolved and natural-evolved *P. aeruginosa* strains. The enhanced biosynthesis of fatty acids reduces membrane permeability and, thereby, inhibits CIP uptake (Figure 9). Therefore, reprogramming metabolomics may be an effective approach to understanding antibiotic-resistant mechanisms and controlling antibiotic-resistant bacteria.

## Data Availability Statement

The original contributions presented in the study are included in the article/Supplementary Material, further inquiries can be directed to the corresponding author.

## Author Contributions

Z-gC conceptualized and designed the project and wrote the manuscript. Z-gC and HL interpreted the data. Y-bS, X-kT, L-pZ, K-xY, and LP performed the data analysis and the experiments. All authors contributed to the article and approved the submitted version.

## Funding

This work was financially supported by grants from the NSFC project (31930115), International Cooperation and Exchanges NSFC (32061133007), the Innovation Group Project of Southern Marine Science and Engineering Guangdong Laboratory (Zhuhai) (311021006), the NSFC project (42076095), and the Natural Science Foundation of Guangdong Province grant (2019A1515012211).

## Conflict of Interest

The authors declare that the research was conducted in the absence of any commercial or financial relationships that could be construed as a potential conflict of interest.

## Publisher's Note

All claims expressed in this article are solely those of the authors and do not necessarily represent those of their affiliated organizations, or those of the publisher, the editors and the reviewers. Any product that may be evaluated in this article, or claim that may be made by its manufacturer, is not guaranteed or endorsed by the publisher.

## Supplementary Material

Supplementary Figure 1 Analysis of *mexCD-oprJ* expression in PA-R. **(A)** qRT-PCR for expression of *mexCD-oprJ* in PA-S0, PA-R16_CIP_, PA-R64_CIP_, and PA-R128_CIP_. **(B)** qRT-PCR for expression of *mexCD-oprJ* in the presence of triclosan (1 μg/ml) in PA-R16_CIP_, PA-R64_CIP_, and PA-R128_CIP_. **(C)** qRT-PCR for expression of *mexCD-oprJ* in the presence of triclosan (1 μg/ml) in clinical *P. aeruginosa* A2 and B2. Results are shown as mean ± SEM and at least three biological repeats are performed. Significant differences are identified ^*^*p* < 0.05, ^**^*p* < 0.01.

Supplementary Figure 2 Triclosan inhibits expression of genes encoding biosynthesis of fatty acids. **(A)** qRT-PCR for expression of genes encoding fatty acid biosynthesis in the presence of triclosan (1 μg/ml) in PA-R16_CIP_, PA-R64_CIP_, and PA-R128_CIP_. **(B)** Activity of ACC in the presence of triclosan (1 μg/ml) in PA-R16_CIP_, PA-R64_CIP_, and PA-R128_CIP_. **(C)** qRT-PCR for expression of genes encoding fatty acid degradation in the presence of triclosan (1 μg/ml) in PA-R16_CIP_, PA-R64_CIP_, and PA-R128_CIP_. **(D)** qRT-PCR for expression of genes encoding fatty acid biosynthesis in the presence of triclosan (1 μg/ml) in clinical *P. aeruginosa* A2 and B2. **(E)** Activity of ACC in the presence of triclosan (1 μg/ml) in clinical *P. aeruginosa* A2 and B2. **(F)** qRT-PCR for expression of genes encoding fatty acid degradation in the presence of triclosan (1 μg/ml) and in clinical *P. aeruginosa* A2 and B2. Results are displayed as mean ± SEM and three biological repeats are performed. Significant differences are identified ^*^*p* < 0.05, ^**^*p* < 0.01.

Supplementary Table 1 Primers for qRT-PCR.
