# Supplementary figures and images for: Enhanced Biosynthesis of Fatty Acids Contributes to Ciprofloxacin Resistance in Pseudomonas aeruginosa
Source: Front Microbiol. 2022 Apr 25;13:845173. doi: 10.3389/fmicb.2022.845173 (PMC9083408; doi:10.3389/fmicb.2022.845173)

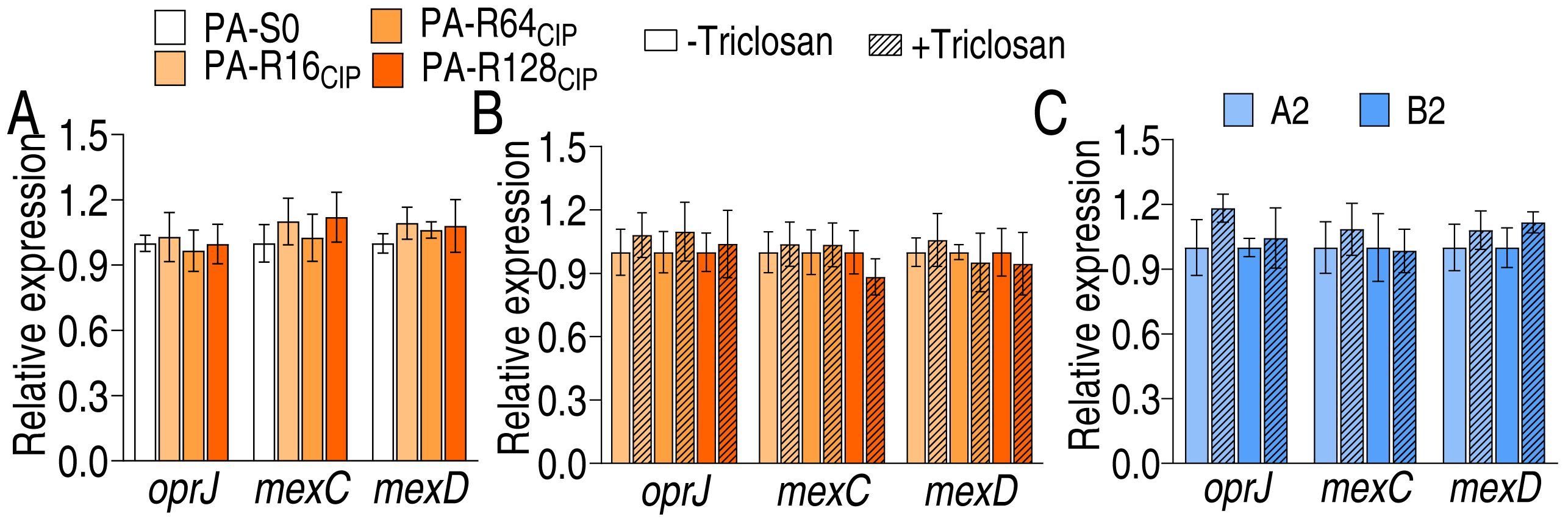

Supplement: Supplementary Figure 1 — Analysis of mexCD-oprJ expression in PA-R. (A) qRT-PCR for expression of mexCD-oprJ in PA-S0, PA-R16CIP, PA-R64CIP, and PA-R128CIP. (B) qRT-PCR for expression of mexCD-oprJ in the presence of triclosan (1 μg/ml) in PA-R16CIP, PA-R64CIP, and PA-R128CIP. (C) qRT-PCR for expression of mexCD-oprJ in the presence of triclosan (1 μg/ml) in clinical P. aeruginosa A2 and B2. Results are shown as mean ± SEM and at least three biological repeats are performed. Significant differences are identified *p < 0.05, **p < 0.01. [file Image_1.TIF]

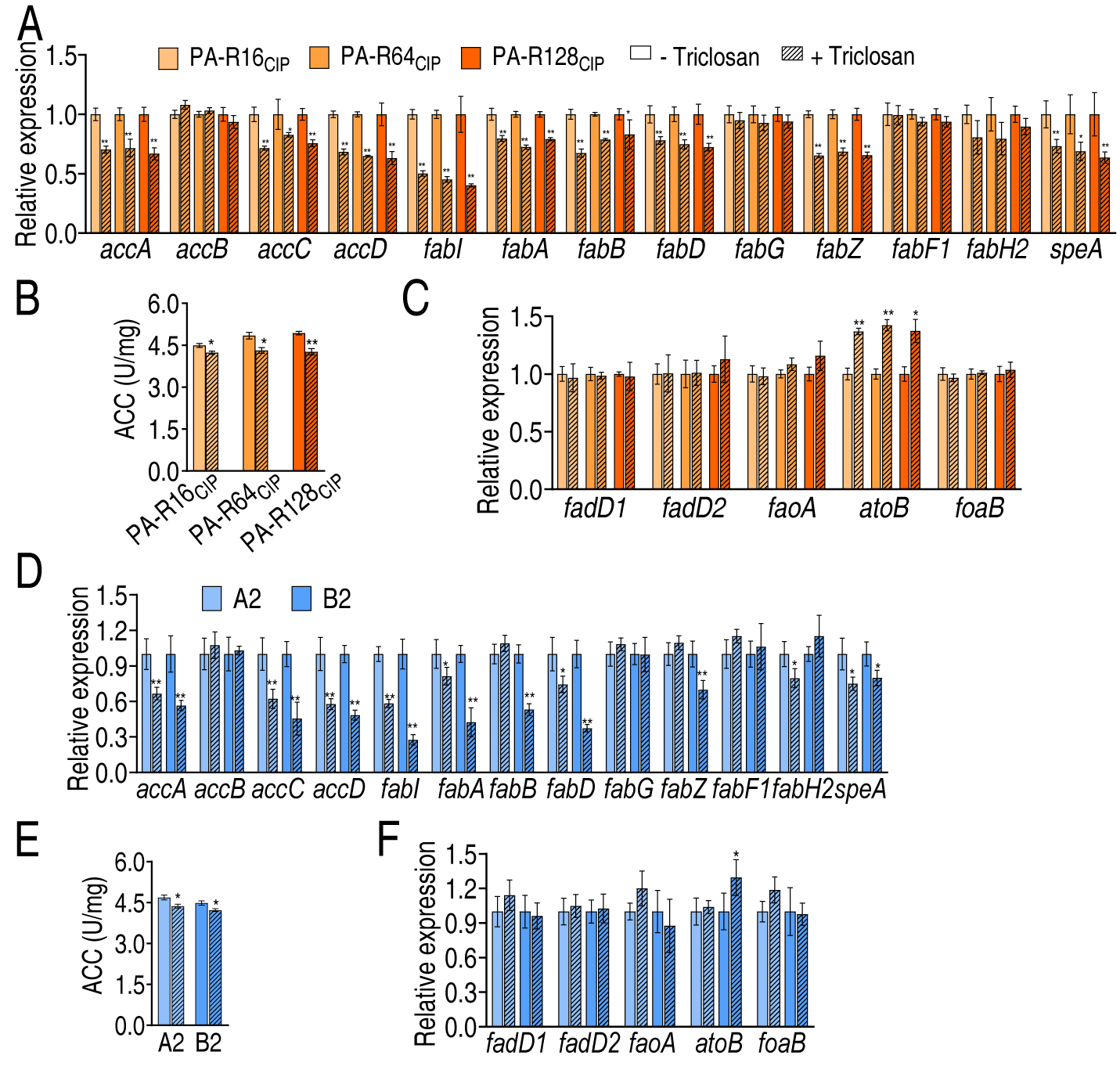

Supplement: Supplementary Figure 2 — Triclosan inhibits expression of genes encoding biosynthesis of fatty acids. (A) qRT-PCR for expression of genes encoding fatty acid biosynthesis in the presence of triclosan (1 μg/ml) in PA-R16CIP, PA-R64CIP, and PA-R128CIP. (B) Activity of ACC in the presence of triclosan (1 μg/ml) in PA-R16CIP, PA-R64CIP, and PA-R128CIP. (C) qRT-PCR for expression of genes encoding fatty acid degradation in the presence of triclosan (1 μg/ml) in PA-R16CIP, PA-R64CIP, and PA-R128CIP. (D) qRT-PCR for expression of genes encoding fatty acid biosynthesis in the presence of triclosan (1 μg/ml) in clinical P. aeruginosa A2 and B2. (E) Activity of ACC in the presence of triclosan (1 μg/ml) in clinical P. aeruginosa A2 and B2. (F) qRT-PCR for expression of genes encoding fatty acid degradation in the presence of triclosan (1 μg/ml) and in clinical P. aeruginosa A2 and B2. Results are displayed as mean ± SEM and three biological repeats are performed. Significant differences are identified *p < 0.05, **p < 0.01. [file Image_2.TIF]
